# Role of Genetic Polymorphisms in the Development and Prognosis of Sporadic and Familial Prostate Cancer

**DOI:** 10.1371/journal.pone.0166380

**Published:** 2016-12-01

**Authors:** Sabrina T. Reis, Nayara I. Viana, Katia R. M. Leite, Erico Diogenes, Alberto A. Antunes, Alexandre Iscaife, Adriano J. Nesrallah, Carlo C. Passerotti, Victor Srougi, José Pontes-Junior, Mary Ellen Salles, William C. Nahas, Miguel Srougi

**Affiliations:** 1 Laboratory of Medical Investigation (LIM55), Urology Department, University of Sao Paulo Medical School, Sao Paulo, Brazil; 2 Uro-Oncology Group, Urology Department, University of Sao Paulo Medical School and Institute of Cancer Estate of Sao Paulo (ICESP), Sao Paulo, Brazil; University of Central Florida, UNITED STATES

## Abstract

**Backgrounds:**

Our aim was to evaluate the role of 20 genetic polymorphisms in the development and prognosis of sporadic and familial PC. A case-control study of 185 patients who underwent radical prostatectomy from 1997 to 2011. These patients were divided into two groups based on their family history. Gleason grade, PSA value and pathological TNM 2002 stage were used as prognostic factors. Blood samples from 70 men without PC were used as controls. The SNPs were genotyped using a TaqMan^®^ SNP Genotyping Assay Kit.

**Results:**

Considering susceptibility, the polymorphic allele in the SNP rs2660753 on chromosome 3 was significantly more prevalent in controls (p = 0.01). For familial clustering, the polymorphic homozygote genotype of the SNP rs7931342 was five times more frequent in patients with familial PC compared to sporadic PC (p = 0.01). Regarding the SNP 1447295, the polymorphic homozygote genotype was more prevalent in patients with organ-confined PC (p = 0.05), and most importantly, the polymorphic allele occurred more frequently in patients without biochemical recurrence (p = 0.01). Kaplan-Meier analysis showed a median biochemical recurrence free survival of 124.2 compared to 85.6 months for patients with the wild-type allele (p = 0.007).

**Conclusion:**

Our findings provide the evidence for the association of 20 recently highlighted SNPs and their susceptibility, familial clustering, staging, Gleason score and biochemical recurrence of PC. We believe that the association between these SNPs and PC may contribute to the development of alternative tools that can facilitate the early detection and prognosis of this disease.

## Introduction

Prostate cancer (PC) is the most commonly diagnosed non-skin tumor in men and is the second leading cause of death in Western countries, including Brazil [1]. Its incidence varies widely between different regions of the world, with the highest rates found in developed countries, which reflect screening practices with prostate specific antigen (PSA) and subsequent biopsy. However, the number of deaths by PC does not vary significantly between developed and developing countries, and this discrepancy is attributed to the increased diagnosis of indolent disease due to PSA screening [2].

Established risk factors for PC include older age, African descent and family history [3]. Heredity is one of the main risk factors of PC, which is characterized by the inheritance of highly penetrant mutations in susceptibility genes [4, 5].

Several studies have shown that approximately 10 to 20% of patients with PC have a positive family history, thus increasing the risk for a man to develop cancer throughout his life by 11 times [6–8]. This risk is even higher when cases are diagnosed in men less than 60 years of age and when more than one relative is affected [9–12]. Currently, we can distinguish three epidemiological forms of PC: (1) sporadic, which occurs randomly in the population; (2) familiar, which is characterized by unpredictable grouping of PC in families; and (3) hereditary, which is characterized by strong clusters and an early onset of cancer.

By studying the human genome, it was possible to infer that the locus genetic variation can be used to identify susceptible families [10]. Association studies and linkage analysis using single nucleotide polymorphisms (SNPs) are interesting to use because they are characterized by having stable inheritance through generations.

Some published studies have associated polymorphisms and susceptibility to PC. Recently, Liu et al [11] performed a meta-analysis combining data from genome-wide association and case-control studies, mostly analyzing men in North America and Europe, and found 31 SNPs in 14 independent risk loci that impacted the susceptibility of PC. Based on this study, we selected 20 SNPs that were relevant to the development of PC in some populations to search for their impact on Brazilian men, who can be characterized as a highly diverse population, based on their genetic profiles, that has a high incidence of PC, including the familial form.

## Materials and Methods

### Patients

This is a case-control study of 185 patients who underwent radical prostatectomy from 1997 to 2011. The same surgeon operated on all patients and, after surgery, were followed in a private clinic with semestral PSA measurement for the first 5 years and then annually for the subsequent 5 years.

Patients were divided into two groups according to the family history: 1, familial PC or 2, individual with sporadic PC. Familial PC was characterized by the presence of two or more first-degree relatives who were also affected by the disease.

The Gleason grade, PSA value and pathological TNM 2010 stage were used as prognostic factors. For analysis, the pathological stage was considered organ-confined (pT2) or non-organ-confined (pT3) disease. The Gleason score was classified as low- or high-grade disease (Gleason score ≤6 and Gleason score ≥7, respectively) and as low-, intermediate- and high-grade (Gleason score ≤6, 7, and > 8, respectively). Preoperative PSA was also used to identify patients who were at high risk (≥10 ng/mL) and low risk (<10 ng/mL) for disease recurrence. The subjects were followed for a mean time of 60 months, and biochemical recurrence was considered when their PSA levels were higher than 0.2 ng/mL.

Seventy men served as controls. Their blood PSA levels were within the normal limit (< 2.5 ng/ml), digital examination of the prostate was unremarkable, and none of them had a family history of PC.

Subjects in both groups provided written informed consent to participate the study and allowed their biological samples to be genetically analyzed. Approval for the study was given by the Institutional Board of Ethics (CAPPesq–Comissão de Ética para Análise de Projetos de Pesquisa) under the number 477/11.

### Analysis of SNPs

Genomic DNA was extracted from blood using QIAmp DNA mini kit (QIAGEN). The SNPs were genotyped using a TaqMan^®^ SNP Genotyping Assay Kit and an ABI 7500 fast system (Applied Biosystems, CA, USA).

SNP-specific polymerase chain reaction (ss-PCR) primers (Table 1) and fluorogenic probes were designed using Primer Express (Applied Biosystems, CA, USA). The fluorogenic probes were labeled with a reporter dye (FAM or VIC) and were specific for one of the two possible bases identified for that site in the gene sequence.

**Table 1:** Primers used in the genotyping of SNPs.

db SNP	Gene/Region	Chromosome	Location	Major allele frequency*	Minor allele frequency*	MAF**
**rs10090154**	8q24	8	Chr.8:128532137	0.94	0.06	0.13
**rs1016343**	8q24	8	Chr.8:128093297	0.74	0.26	0.20
**rs1859962**	17q24.3	17	Chr.17:69108753	0.53	0.47	0.42
**rs1447295**	8q24	8	Chr.8:128485038	0.93	0.07	0.18
**rs16901979**	8q24	8	Chr.8:128124912	0.97	0.03	0.21
**rs2660753**	3P12.1	3	Chr.3:87110674	0.90	0.10	0.29
**rs2710646**	EHBP1	2	Chr.2:63134879	0.86	0.14	0.09
**rs3760511**	17q12	17	Chr.17:36106313	0.73	0.27	0.39
**rs4242382**	8q24	8	Chr.8:128517573	0.93	0.07	0.18
**rs4962416**	CTBP2	10	Chr.10:126696872	0.74	0.26	0.18
**rs5945619**	NUDT11	X	Chr.X:51241672	0.61	0.39	0.27
**rs620861**	-	8	Chr.8:128335673	0.62	0.38	0.40
**rs6501455**	17q24.3	17	Chr.17:69201811	0.54	0.46	0.34
**rs6983267**	8q24	8	Chr.8:128413305	0.51	0.49	0.39
**rs6983561**	8q24	8	Chr.8:128106880	0.97	0.03	0.21
**rs7000448**	8q24	8	Chr.8:128441170	0.64	0.36	0.41
**rs7214479**	17q24.3	17	Chr.17:69190949	0.58	0.42	0.39
**rs7920517**	MSMB	10	Chr.10:51532621	0.58	0.42	0.42
**rs7931342**	11q13.2	11	Chr.11:68994497	0.53	0.47	0.48
**rs983085**	17q24.3	17	Chr.17:983085	0.53	0.47	0.48

* Caucasian population

**Global MAF

The target sequence was amplified in a 10 μl reaction volume that contained 5 μl of TaqMan^®^ Universal PCR Master Mix, 0.25 μl of SNP Genotyping Assay (primers and probes), 1 μl of genomic DNA, and 3.75 μl of DNase-free water. The PCR cycling conditions were 2 minutes at 50°C and 10 minutes at 95°C, followed by 40 cycles of 15 seconds at 95°C and 60 seconds at 60°C. After PCR amplification, an endpoint plate reading was performed using an Applied Biosystems 7500 fast Real-Time PCR System. The Sequence Detection System (SDS) software uses the fluorescence measurements made during the plate reading to plot fluorescence (Rn) values based on the signals from each well. The plotted fluorescence signals indicate which alleles are in each sample.

### Statistical analysis

Categorical variables were express as number and percentage. The associations between genotype and allelic frequencies in the cases and controls were examined by the Fisher exact odds ratio (OR) and the corresponding 95% confidence intervals (CIs) were provided. To compare the clinical characteristics of patients with PC, we used the chi-square test. Statistical analysis were perform using SPSS 19.0 for Windows, and significance was identified at p ≤0.05.

## Results

Table 2 shows the clinical characteristics of the patients. The genotypic distributions among PC patients and controls are shown in Table 3. They did not significantly deviate from the values expected for the Hardy-Weinberg equilibrium.

**Table 2:** Clinical and pathological characteristics of patients with PC and the control group.

	Prostate cancer Mean (SD*)	Control group Mean (SD*)	p
Age (years)	60.23 (8.05)	67.24 (8.87)	0.00
PSA (ng/ml)	8.38 (11.20)	1.17 (0.82)	0.00
Volume (gr)	40.92 (16.39)	34.62 (13.67)	0.00

*SD = standard deviation

**Table 3:** Allele and genotype frequencies in the prostate cancer and control groups.

ID SNP	Genotype	PC (n)	Control (n)	OR	p	Allele	PC	Control	OR	p
rs4242382										
	GG*	36.4% (59)	43.9% (29)	1.00	0.32	G*	26.4%	33.0%	1.00	0.29
	GA	14.8% (24)	18.2% (12)	0.93 [0.40–2.13]		A	73.6%	67.0%	1.36 [0.76–2.44]	
	AA	48.8% (79)	37.9% (25)	1.47 [0.78–2.78]						
rs10090154										
	CC*	80.0% (136)	79.1% (53)	1.00	0.97	C*	29.2%	27.9%	1.00	0.86
	CT	18.8% (32)	19.4% (13)	0.93 [0.45–1.91]		T	70.8%	72.1%	0.94 [0.46–1.88]	
	TT	1.2% (2)	1.5% (1)	0.75 [0.06–8.55]						
rs1016343										
	CC*	77.4% (123)	84.1% (53)	1.00	0.43	C*	21.7%	30.1%	1.00	0.26
	CT	18.2% (29)	14.3% (9)	1.35 [0.59–3.05]		T	78.3%	69.9%	1.55 [0.71–3.35]	
	TT	4.4% (7)	1.6% (1)	2.93 [0.35–24.4]						
rs1447295										
	CC*	43.2% (73)	40.9% (27)	1.00	0.94	C*	28.9%	27.0%	1.00	0.75
	CA	17.2% (29)	18.2% (12)	0.84 [0.37–1.90]		A	71.1%	73.0%	0.91 [0.51–1.62]	
	AA	39.6% (67)	40.9% (27)	0.87 [0.46–1.64]						
rs16901979										
	CC*	64.7% (55)	58.8% (20)	1.00	0,65	C*	31.1%	27.0%	1.00	0.63
	CA	4.7% (4)	8.8% (3)	0.45 [0.09–2.20]		A	68.9%	73.0%	0.82 [0.36–1.85]	
	AA	30.6% (26)	32.4% (11)	0.80 [0.33–1.92]						
rs2660753										
	CC*	62.3% (109)	45.5% (30)	1.00	**0.05**	C*	35.3%	21.6%	1.00	**0.01**
	CT	33.1% (58)	50.0% (33)	0.50 [0.28–0.91]		T	64.7%	78.4%	0.50 [0.28–0.89]	
	TT	4.6% (8)	4.5% (3)	0.73 [0.18–2.93]						
rs2710646										
	CC*	35.8% (62)	41.2% (28)	1.00	0.17	C*	26.5%	31.1%	1.00	0.44
	CA	26.0% (45)	14.7% (10)	1.92 [0.84–4.38]		T	73.5%	68.9%	1.25 [0.70–2.22]	
	AA	38.2% (66)	44.1% (30)	0.94 [0.50–1.76]						
rs3760511										
	TT*	8.6% (15)	6.0% (4)	1.00	0.79	T*	28.3%	21.1%	1.00	0.50
	TG	84.0% (147)	86.6% (58)	0.69 [0.22–2.17]		G	71.7%	78.9%	0.67 [0.21–2.11]	
	GG	7.4% (13)	7.5% (5)	0.69 [0.15–3.13]						
rs4962416										
	TT*	12.0% (20)	13.4% (9)	1.00	0.87	T*	28.4%	31.0%	1.00	0.77
	TC	75.3% (125)	76.1% (51)	1.13 [0.48–2.65]		C	71.6%	69.0%	1.13 [0.48–2.63]	
	CC	12.7% (21)	10.4% (7)	1.35 [0.42–4.31]						
rs5945619										
	TT*	7.8% (13)	6.7% (4)	1.00	0.12	T*	26.8%	23.5%	1.00	0.76
	TC	88.0% (146)	81.7% (49)	0.94 [0.29–3.02]		C	73.2%	76.5%	0.84 [0.26–2.68]	
	CC	4.2% (7)	11.7% (7)	0.30 [0.06–1.42]						
rs620861										
	CC*	27.4% (48)	22.4% (15)	1.00	0.69	C*	29.1%	23.8%	1.00	0.42
	CT	40.0% (70)	44.8% (30)	0.76 [0.37–1.57]		T	70.9%	76.2%	0.76 [0.39–1.48]	
	TT	32.6% (57)	32.8% (22)	0.81 [0.37–1.73]						
rs6501455										
	GG*	30.9% (46)	25.0% (15)	1.00	0.25	G*	30.4%	24.6%	1.00	0,39
	GA	52.3% (78)	48.3% (29)	0.92 [0.44–1.89]		A	69.6%	75.4%	0.74 [0.37–1.47]	
	AA	16.8% (25)	26.7% (16)	0.51 [0.21–1.20]						
rs6983267										
	GG*	6.8% (11)	3.1% (2)	1.00	0.44	G*	29.4%	15.4%	1.00	0.27
	GT	92.6% (150)	95.4% (62)	0.45 [0.09–2.09]		T	70.6%	84.6%	0.43 [0.09–2.02]	
	TT	0.6% (1)	1.5% (1)	0.18 [0.08–4.26]						
rs6983561										
	AA*	81.0% (128)	82.5% (52)	1.00	0.93	A*	26.8%	28.9%	1.00	0.79
	AC	17.7% (28)	15.9% (10)	1.10 [0.50–2.44]		C	73.2%	71.1%	1.10 [0.51–2.37]	
	CC	1.3% (2)	1.6% (1)	0.79 [0.07–8.91]						
rs7000448										
	GG*	29.8% (53)	28.4% (19)	1.00	0.06	G*	27.7%	26.4%	1.00	0.82
	GA	55.6% (99)	67.2% (45)	0.81 [0.43–1.53]		A	72.3%	73.6%	0.93 [0.50–1.73]	
	AA	14.6% (26)	4.5% (3)	3.10 [0.82–8.91]						
rs7214479										
	CC*	1.1% (2)	0 (0)	-	0.68	C*	26.9%	0%	1.00	0.54
	CT	95.5% (170)	96.9% (63)	-		T	73.1%	100%	-	
	TT	3.4% (6)	3.1% (2)	-						
rs7920517										
	AA*	6.8% (12)	2.9% (2)	1.00	0.40	A*	28.6%	14.3%	1.00	0.24
	AG	65.0% (115)	72.1% (49)	0.40 [0.08–1.86]		G	71.4%	85.7%	0.41 [0.09–1.91]	
	GG	28.2% (50)	25.0% (17)	0.49 [0.09–2.41]						
rs7931342										
	GG*	27.3% (48)	29.4% (20)	1.00	0.88	G*	27.3%	29.4%	1.00	0.73
	GT	67.0% (118)	66.2% (45)	1.12 [0.60–2.10]		T	72.7%	70.6%	1.11 [0.59–2.06]	
	TT	5.7% (10)	4.4% (3)	1.38 [0.34–5.58]						
rs983085										
	AA*	36.0% (63)	29.4% (20)	1.00	0.19	A*	30.0%	24.1%	1.00	0.33
	AG	46.3% (81)	42.6% (29)	0.93 [0.48–1.80]		G	70.0%	75.9%	0.74 [0.40–1.35]	
	GG	17.7% (31)	27.9% (19)	0.51 [0.24–1.10]						
rs1859962										
	TT*	18.8% (33)	10.9% (7)	1.00	0.25	T*	28.6%	17.1%	1.00	0.12
	TG	69.3% (168)	71.9% (46)	0.58 [0.23–1.40]		G	71.4%	82.9%	0.51 [0.21–1.22]	
	GG	11.9% (21)	17.2% (11)	0.40 [0.13–1.21]						

*wild-type

There was a statistically significant difference in the distribution of the genotypes between patients and controls for the SNP rs2660753. The frequencies of the CC, TT, and CT genotypes were 45.5%, 4.5%, and 50.0% in healthy controls and 62.3%, 4.6%, and 33.1% in PC patients, respectively (p<0.05). There were also statistically significant differences in the allelic frequencies between PC patients and controls for this SNP. The C and T alleles were detected in 35.3% and 64.7% of healthy controls and in 21.6% and 78.4% of PC patients, respectively (p = 0.01). These results are shown in Table 3.

For familial clustering, we found a significant association for the SNP rs7931342, in which the polymorphic homozygote genotype was four times more common in patients with familial PC than in patients with sporadic PC (p = 0.01) (Table 4). For allelic frequencies, we found statistically significant differences for the SNPs rs10090154 and rs7000448. For the SNP rs10090154, we found that when patients had the polymorphic allele, the rate of PC with familial clustering was 61.3%; the rate of sporadic PC was 62.8% in patients with the wild-type allele. Thus, the presence of the polymorphic allele increased the chance of familial PC by 2.5 times (p = 0.01). For the SNP rs7000448, we found that the wild-type allele occurred more frequently in patients with familial PC and that the polymorphic allele was more frequent in patients with sporadic PC (OR = 0.5, p = 0.04) (Table 4).

**Table 4:** Allele and genotype frequencies in sporadic (S) prostate cancer and familial (F) prostate cancer.

ID SNP	Genotype	S PC (n)	F PC (n)	OR	p	Allele	S PC	F PC	OR	p
rs4242382										
	GG+	42.9% (36)	32.4% (22)	1.00	0.29	G*	61.0%	51.6%	1.00	0.25
	GA	11.9% (10)	19.1% (13)	2.12 [0.79–5.67]		A	39.0%	48.4%	1.46 [0.75–2.84]	
	AA	45.2% (38)	48.5% (33)	1.42 [0.70–2.88]						
rs10090154										
	CC*	86.0% (80)	71.6% (48)	1.00	**0.03**	C*	62.8%	38.7%	1.00	**0.01**
	CT	14.0% (13)	25.4% (17)	2.17 [0.97–4.87]		T	37.2%	61.3%	2.67 [1.19-5-98]	
	TT	0 (0)	3.0% (2)	-						
rs1016343										
	CC*	74.1% (63)	81.3% (52)	1.00	0.54	C*	54.4%	65.7%	1.00	0.23
	CT	20.0% (17)	15.6% (10)	0.71 [0.30–1.68]		T	45.6%	34.3%	0.62 [0.28–1.37]	
	TT	5.9% (5)	3.1% (2)	0.48 [0.09–1.68]						
rs1447295										
	CC*	49.4% (44)	40.0% (28)	1.00	0.48	C*	61.1%	51.7%	1.00	0.23
	CA	16.9% (15)	18.6% (13)	1.36 [0.56–3.28]		A	38.9%	48.3%	1.46 [0.77–2.76]	
	AA	33.7% (30)	41.4% (29)	1.51 [0.75–3.04]						
rs16901979										
	CC*	73.3% (33)	57.9% (22)	1.00	0.31	C*	60.0%	42.9%	1.00	0.13
	CA	4.4% (2)	5.3% (2)	1.50 [0.19–11.4]		A	40.0%	57.1%	2.00 [0.42–1.51]	
	AA	22.2% (10)	36.8% (14)	2.10 [0.79–5.56]						
rs2660753										
	CC*	59.6% (56)	64.8% (46)	1.00	0.77	C*	54.9%	60.3%	1.00	0.49
	CT	36.2% (34)	31.0% (22)	0.78 [0.40–1.52]		T	45.1%	39.7%	0.81 [0.42–1.52]	
	TT	4.3% (4)	4.2% (3)	0.91 [0.19–4.28]						
rs2710646										
	CC*	41.7% (40)	30.9% (21)	1.00	0.17	C*	65.6%	54.4%	1.00	0.15
	CA	28.1% (27)	25.0% (17)	1.19 [0.53–2.68]		A	34.4%	45.6%	1.59 [0.83–3.07]	
	AA	30.2% (29)	44.1% (30)	1.97 [0.94–4.10]						
rs3760511										
	TT*	8.4% (8)	8.6% (6)	1.00	0.34	T*	53.8%	57.9%	1.00	0.77
	TG	86.3% (82)	80.0% (56)	0.91 [0.30–2.78]		G	46.2%	42.1%	0.84 [0.27–2.64]	
	GG	5.3% (5)	11.4% (8)	2.13 [0.45–9.94]						
rs4962416										
	TT*	13.2% (12)	12.3% (8)	1.00	0.42	T*	60.0%	58.1%	1.00	0.87
	TC	72.5% (66)	80.0% (52)	1.18 [0.45–3.10]		C	40.0%	41.9%	1.08 [0.41–2.81]	
	CC	14.3% (13)	7.7% (5)	0.57 [0.14–2.26]						
rs5945619										
	TT*	9.8% (9)	4.4% (3)	1.00	0.34	T*	75.0%	56.1%	1.00	0.20
	TC	87.0% (80)	89.7% (61)	2.28 [0.59–8.81]		C	25.0%	43.9%	2.34 [0.61–9.03]	
	CC	3.3% (2)	5.9% (4)	4.00 [0.54–29.1]						
rs620861										
	CC*	27.1% (26)	27.5% (19)	1.00	0.51	C*	57.8%	58.3%	1.00	0.94
	CT	37.5% (36)	44.9% (31)	1.17 [0.55–2.52]		T	42.2%	41.7%	0.97 [0.48–1.95]	
	TT	35.4% (34)	27.5% (19)	0.76 [0.33–1.72]						
rs6501455										
	GG*	33.3% (28)	29.8% (17)	1.00	0.45	G*	62.2%	58.3%	1.00	0.66
	GA	53.6% (45)	49.1% (28)	1.02 [0.47–2.20]		A	37.8%	41.7%	1.17 [0.56–2.43]	
	AA	13.1% (11)	21.1% (12)	1.79 [0.65–4.96]						
rs6983267										
	GG*	8.1% (7)	6.1% (4)	1.00	0.59	G*	83.3%	55.5%	1.00	0.17
	GT	90.7% (78)	93.9% (62)	1.39 [0.39–4.96]		T	16.7%	44.5%	4.01 [0.45–35.20]	
	TT	1.2% (1)	0 (0)	-						
rs6983561										
	AA*	79.1% (68)	83.1% (54)	1.00	0.77	A*	55.4%	63.3%	1.00	0.43
	AC	19.8% (17)	15.4% (10)	0.74 [0.31–1.74]		C	44.6%	36.7%	0.71 [0.31–1.63]	
	CC	1.2% (1)	1.5% (1)	1.25 [0.07–20.5]						
rs7000448										
	GG*	24.7% (24)	39.4% (28)	1.00	0.10	G*	46.2%	62.9%	1.00	**0.04**
	GA	57.7% (56)	49,3% (35)	0.53 [0.26–1.06]		A	53.8%	37.1%	0.50 [0.26–0.98]	
	AA	17.5% (17)	11,3% (8)	0.40 [0.14–1.09]						
rs7214479										
	CC*	1.0% (1)	1.4% (1)	1.00	0.14	C*	50.0%	57.2%	1.00	0.83
	CT	93.8% (90)	98.6% (71)	0.78 [0.04–12.83]		T	50.0%	42.8%	0.74 [0.04–12.15]	
	TT	5.2% (5)	0 (0)	-						
rs7920517										
	AA*	7.3% (7)	5,6% (4)	1.00	0.77	A*	63.6%	57.1%	1.00	0.66
	GG	30.2% (29)	26,8% (19)	1.40 [0.38–5.06]		G	36.4%	42.9%	1.31 [0.37–4.68]	
	AG	62.5% (60)	67,6% (48)	1.14 [0.29–4.45]						
rs7931342										
	GG*	24.2% (23)	32.4% (23)	1.00	**0.01**	G*	53.8%	57.9%	1.00	0.33
	GT	73.7% (70)	56.3% (40)	0.57 [0.28–1.14]		T	46.2%	42.1%	0.84 [0.27–2.64]	
	TT	2.1% (2)	11.3% (8)	4.00 [1.01–20.9]						
rs983085										
	AA*	36.6% (34)	36.1% (26)	1.00	0.99	A*	56.7%	56.2%	1.00	0.95
	AG	46.2% (43)	45.8% (33)	1.00 [0.50–1.98]		G	43.3%	43.8%	1.02 [0.53–1.93]	
	GG	17.2% (16)	18.1% (13)	1.06 [0.43–2.59]						
rs1859962										
	TT*	18.1% (17)	20.8% (15)	1.00	0.69	T*	53.1%	57.5%	1.00	0.65
	TG	68.1% (64)	69.4% (50)	0.88 [0.40–1.94]		G	46.9%	42.5%	0.83 [0.38–1.82]	
	GG	13.8% (13)	9.7% (7)	0.61 [0.19–1.93]						

*wild-type

An additional analysis was performed according to the PSA value, pathological stage and Gleason score to evaluate differences in prognosis (Table 5 and S1 Table). For the PSA value, there were differences for the SNP rs6983267; although most of the patients had the heterozygote genotype, no patients with PSA≥ 10 ng/ml had a wild-type homozygote genotype, and no patient with PSA < 10 ng/ml had a polymorphic homozygote genotype (p = 0.02).

**Table 5:** Genotype frequencies according to prognostic factors.

ID SNP	Genotype	Prognostic Factors		OR	p
		**PSA < 10 ng/ml (n)**	**PSA ≥ 10 ng/ml (n)**		
rs6983267	GG	8.1% (11)	0% (0)	**-**	**0.02**
	GT	91.5% (118)	95.8% (23)	**-**	
	TT	0% (0)	4.2% (1)	**-**	
		**pT2 (n)**	**pT3 (n)**		
rs1447295	CC	43.9% (50)	64.0% (16)	1.00	**0.05**
	CA	14.9% (17)	20.0% (5)	0.91 [0.29–2.88]	
	AA	41.2% (47)	16.0% (4)	0.26 [0.08–0.85]	
		**Gleason ≤ 6**	**Gleason 7**	**Gleason ≥8**	
rs7931342	GG	29.3% (12)	33.3% (18)	17.9% (10)	**0.02**
	GT	70.7% (29)	63.0% (34)	67.9% (38)	
	TT	0 (0)	3.7% (2)	14.3% (8)	

Analyzing the pathological stage and genotype distributions, we found significant differences in the SNP rs1447295. The wild-type homozygote genotype was more frequent in non–organ-confined tumors (pT3) (p = 0.05).

For the Gleason score, different analysis were performed. We observed differences for the SNP rs7931342 when we compared this SNP using the three Gleason categories. We found that the incidence of the polymorphic homozygote genotype was significantly higher in patients with a Gleason score ≥ 8 (p = 0.02). No statistical association was observed between the Gleason score and the other SNPs (S2 Table).

Our analysis of biochemical recurrence is shown in Table 6. We found a frequency of 71.0% for the SNP rs1447295 in the wild-type homozygote genotype in patients with recurrence, whereas the rate was 42.7% for patients without this profile, (p = 0.01). There were also significant differences in the allelic frequencies for biochemical recurrence for this SNP. In the same way, the wild-type allele was more frequent in patients with recurrence compared to patients without (p = 0.00). For the SNP rs7214479, all patients without biochemical recurrence had the polymorphic allele, but only 21.4% of patients with recurrence had the same profile (p = 0.005; Table 6) (S3 Table).

**Table 6:** Genotype and allele frequencies according to biochemical recurrence.

ID SNP		Biochemical recurrence (n)		OR	p
rs1447295	Genotype	Yes	no		
	CC	42.7% (50)	71.0% (22)	1.00	**0.01**
	CA	17.9% (21)	6.5% (2)	0.21 [0.04–1.00]	
	AA	39.3% (46)	22.6% (7)	0.34 [0.13–0.88]	
	Allele				
	C*	69.4%	88.2%	1.00	**0.00**
	A	30.6%	11.8%	0.30 [0.12–0.72]	
rs7214479	Genotype	Yes	no		
	CC	0% (0)	2.9% (1)		0.16
	CT	95.9% (116)	94.1% (32)	-	
	TT	4.1% (5)	2.9% (1)	-	
	Allele				
	C*	0%	78.6%	1.00	**0.05**
	T	100%	21.4%	-	

*wild-type

**Fig 1:**
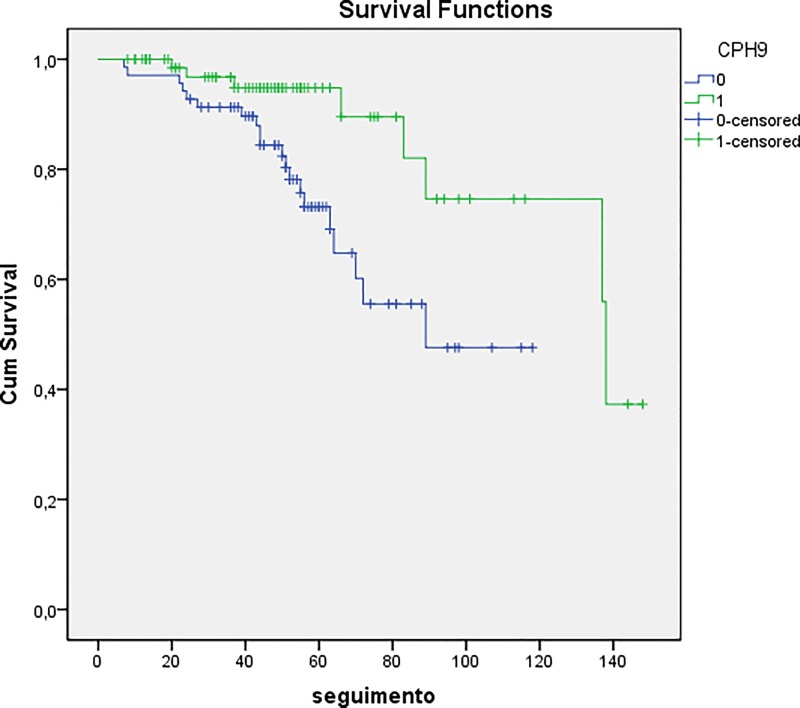
Kaplan-Meier analysis of biochemical recurrence-free survival according to SNP rs1447295. The median biochemical recurrence free survival was 124.2 months for patients with polymorphic allele (1 –green line) vs. 85.6 months for patients with the wild-type allele (0 –blue line).

Kaplan-Meier curve shows that the SNP rs1447295 was related to biochemical recurrence after radical prostatectomy. The median biochemical recurrence free survival time was 124.2 months for patients harboring the polymorphic allele compared to 85.6 months for patients with a wild-type allele (p = 0.007; Fig 1). All patients information and genotyping are shown in S1 Data.

## Discussion

In this case-control study, we investigated the SNPs associated with familial clusters and the prognosis of patients with PC. Considering the development of PC, we found that the polymorphic allele and genotype of the SNP rs2660753 were significantly more prevalent in controls, which suggests that these polymorphisms may play a protective role against the development of PC in our population. The SNP rs2660753 is a PC susceptibility polymorphism that was identified in a genome-wide association study in Europeans and has been validated for different populations of Europeans [12] and non-Europeans [13]. The nearest genes (70–198 kb away) to the region rs2660753 are *VGLL3*, *CHMP2B* and *Pit-1*/*POU1F1*, which encode proteins with potential roles in tumorigenesis. VGLL3 (f70 kb away) encodes a colon carcinoma-related protein. *POU1F1* (198 kb away) encodes POU domain class 1 transcription factor 1. *POU1F1* is a pituitary-specific transcription factor that is centrally involved in regulating growth hormone (GH) synthesis. It is expressed in normal and human breast tumors and regulates GH secretion and cell proliferation. *CHMP2B* (166 kb away) encodes chromatin-modifying protein 2B. CHMP2B belongs to the chromatin-modifying protein/charged multivesicular body protein family. In addition, the 3p12.3-pcen region has been identified as a locus harboring candidate to a tumor suppressor gene [14]. However, the same SNP rs2660753 on chromosome 3 was not correlated with PC in Chinese patients [15].

Considering familial clusters, we found that the polymorphic homozygote genotype of the SNP rs7931342 was five times more prevalent in patients with familial clustering than in patients with sporadic PC. The SNP rs7931342 is a *G/T* variation located on human chromosome 11, which was first reported in early-onset and familial PC by GWAS [16] and subsequently confirmed in another study published by the PRACTICAL consortium with 7,370 PC cases and 5,742 controls [12]. We did not observe the same result for allelic frequencies, possibly because it is necessary to occur in two mutant alleles. For this same SNP, we found an association for the Gleason score. No patient with a Gleason score ≤ 6 had the polymorphic homozygote genotype; it was more frequent in patients with a Gleason score ≥ 8. This same SNP has never been associated with the Gleason score. In this study, patients with the genotype *TG* for this SNP were positively associated with an increased Gleason score (P = 0.04, OR = 2.15, 95% CI = 1.02–4.55) [17]. Because these PCa risk-associated loci are located in inter-genic regions, their functions related to PCa are still not clear.

Interestingly, in the Brazilian population, we found other associations for allelic frequencies and familial PC. The polymorphic allele of the SNP rs10090154 was more prevalent in patients with familial PC, increasing the risk by 2.5 times. In contrast, we found that the SNP rs7000448 polymorphic allele was more frequent in patients with sporadic PC. This SNP is located on human chromosome 8q24; this region is a risk locus for many cancers and is currently considered the most important susceptibility region for PC risk. The mechanisms through which 8q24 affects susceptibility to PC remain poorly understood. Evidence has shown that this risk region may function as a regulatory hub by physical interactions with multiple genes important for prostate carcinogenesis such as *PVT1* (a host gene for several miRNAs), *FAM84B* and *GSDMC* [18]. The SNP rs10090154 was included in an International Consortium of Prostate Cancer Genetics, and in a population-based case-control cohort, it was found to be associated with the risk of familial disease, thus confirming our results [19]. However, there are no data in the literature associating the SNP rs7000448 with familial PC.

Considering the prognostic factors, we found some significant associations. Regarding the SNP rs1447295, we found an association with the pathological tumor stage. The polymorphic homozygote genotype was more prevalent in patients with organ-confined PC. Moreover, the allelic polymorphic was more frequent in patients without biochemical recurrence. The Kaplan-Meier analysis showed a median of a biochemical recurrence free survival rate of 124.2 months compared to 85.6 months for patients with the wild-type allele. Zheng et al. [20] studied men with PC in a Swedish population and showed that the SNP rs1447295 from region 1 of chromosome 8q24 was one of the most strongly associated SNP with risk of PC, but it was not associated with the aggressiveness of PC. However, in another study, this same SNP was associated with a significant increased risk for biochemical recurrence. This result should be considered with caution because that study had a limited number of rs1447295 homozygous carriers [21].

## Conclusions

In conclusion, by systematically evaluating 20 recently highlighted prostate cancer susceptibility SNPs, we provide the evidence for the association of these variants and familial clusters of PC, prognostic factors and biochemical recurrence after treatment. The limitation of this study include the small number of samples and the heterogeneity of the Brazilian population, which limits the generalizability of these findings to other ethnic groups. Thus, further functional analyses and large independent studies in other ethnic populations are required to validate the relevance of the observed associations to the susceptibility and behavior of PC. However, we believe that the association between these SNPs and PC could contribute to the development of alternative tools that may allow the early detection and characterization of the prognosis of this disease.

## Supporting Information

S1 Table PSA and Pathological Stage. Genotype frequencies according to PSA level and pathological stage
(DOCX)

S2 Table Gleason Score. Genotype frequencies in Gleason <7 and ≥ 7 and Gleason <7, Gleason 7 and Gleason ≥ 8.
(DOCX)

S3 Table Recurrence. Allele and genotype frequencies according to biochemical Recurrence
(DOCX)

S1 Data Statistic file. All patients information and genotyping.
(XLSX)
