# Supplementary material for: Role of Genetic Polymorphisms in the Development and Prognosis of Sporadic and Familial Prostate Cancer
Source: PLoS One. 2016 Dec 1;11(12):e0166380. doi: 10.1371/journal.pone.0166380 (PMC5132395; doi:10.1371/journal.pone.0166380)
Supplement: S1 Table — Genotype frequencies according to PSA level and pathological stage (DOCX) [file pone.0166380.s001.docx]

**S1 table. PSA and Pathological Stage.** Genotype frequencies according to PSA level and pathological stage

| ID SNP | Genotype | PSA <10 (n) | PSA≥10 (n) | Odds Ratio | p | pT2 (n) | pT3 (n) | Odds Ratio | p |
| --- | --- | --- | --- | --- | --- | --- | --- | --- | --- |
| rs4242382 |  |  |  |  |  |  |  |  |  |
|  | GG* | 36.7% (47) | 41.7% (10) | 1 | 0.79 | 38.3% (41) | 45.8% (11) | 1 | 0.35 |
|  | GA | 14.1% (18) | 16.7% (4) | 1.04 [0.29 – 3.75] |  | 13.1% (14) | 20.8% (5) | 1.33 [0.21 – 1.55] |  |
|  | AA | 49.2% (63) | 41.7% (10) | 0.74 [0.28 – 1.93] |  | 48.6% (52) | 33.3% (8) | 0.57 [0.39 – 4.50] |  |
| rs10090154 |  |  |  |  |  |  |  |  |  |
|  | CC* | 81.3% (109) | 81.5% (53) | 1 | 0.80 | 81.3% (91) | 76.9% (20) | 1 | 0.62 |
|  | CT | 17.2% (23) | 18.5% (5) | 1.07 [0.36 – 3.14] |  | 17.0% (19) | 23.1% (6) | 1.43 [0.50 – 4.05] |  |
|  | TT | 1.5% (2) | 0 (0) | – |  | 1.8% (2) | 0 (0) | – |  |
| rs1016343 |  |  |  |  |  |  |  |  |  |
|  | CC* | 80.2% (101) | 66.7% (16) | 1 | 0.10 | 75.7% (81) | 90.9% (20) | 1 | 0.26 |
|  | CT | 16.7% (21) | 20.8% (5) | 1.50 [0.49 – 4.55] |  | 20.6% (22) | 9.1% (2) | 0.36 [0.08 – 1.69] |  |
|  | TT | 3.2% (4) | 12.5% (3) | 4.73 [0.96 – 23.15] |  | 3.7% (4) | 0 (0) | – |  |
| rs1447295 |  |  |  |  |  |  |  |  |  |
|  | CC* | 45.2% (61) | 42.3% (11) | 1 | 0.84 | 43.9% (50) | 64.0% (16) | 1 | **0,05** |
|  | CA | 14.8% (20) | 19.2% (5) | 1.38 [0.43 – 4.47] |  | 41.2% (47) | 20.0% (5) | 0.31 [0.29 – 2.88] |  |
|  | AA | 40.0% (54) | 38.5% (10) | 1.02 [0.40 – 2.60] |  | 14.9% (17) | 16.0% (4) | 0.26 [0.08 – 0.85] |  |
| rs16901979 |  |  |  |  |  |  |  |  |  |
|  | CC* | 66.2% (47) | 70.0% (7) | 1 | 0,64 | 67.2% (39) | 68.8% (11) | 1 | 0,75 |
|  | CA | 4.2% (3) | 10.0% (1) | 2.23 [0.20 – 24.63] |  | 3.4% (2) | 0 (0) | – |  |
|  | AA | 29.6% (21) | 20.0% (2) | 0.63 [0.12 – 3.34] |  | 29.3% (17) | 31.3% (5) | 1.04 [0.31 – 3.46] |  |
| rs2660753 |  |  |  |  |  |  |  |  |  |
|  | CC* | 64.3% (90) | 55.6% (15) | 1 | 0.62 | 60.7% (71) | 59.3% (16) | 1 | 0.92 |
|  | CT | 31.4% (44) | 37.0% (10) | 1.36 [0.56 – 3.28] |  | 34.2% (40) | 37.0% (10) | 1.10 [0.46 – 2.67] |  |
|  | TT | 4.3% (6) | 7.4% (2) | 2.00 [0.36 – 10.85] |  | 5.1% (6) | 3.7% (1) | 0.74 [0.08 – 6.57] |  |
| rs2710646 |  |  |  |  |  |  |  |  |  |
|  | CC* | 37.0% (51) | 38.5% (10) | 1 | 0.86 | 38.5% (45) | 48.0% (12) | 1 | 0.53 |
|  | CA | 23.2% (32) | 26.9% (7) | 1.11 [0.38 – 3.22] |  | 25.6% (30) | 16.0% (4) | 0.50 [0.14 – 1.69] |  |
|  | AA | 39.9% (55) | 34.6% (9) | 0.83 [0.31 – 2.21] |  | 35.9% (42) | 36.0% (9) | 0.80 [0.30 – 2.10] |  |
| rs3760511 |  |  |  |  |  |  |  |  |  |
|  | TT* | 8.6% (12) | 8.0% (2) | 1 | 0.78 | 7.7% (9) | 3.7% (1) | 1 | 0.75 |
|  | TG | 83.6% (117) | 88.0% (22) | 1.12 [0.23 – 5.39] |  | 84.6% (99) | 88.9% (24) | 2.18 [0.26 – 18.06] |  |
|  | GG | 7.9% (11) | 4.0% (1) | 0.54 [0.04 – 6.88] |  | 7.7% (9) | 7.4% (2) | 2.00 [0.15 – 26.18] |  |
| rs4962416 |  |  |  |  |  |  |  |  |  |
|  | TT* | 12.2% (16) | 7.7% (2) | 1 | 0.77 | 12.6% (14) | 7.7% (2) | 1 | 0.56 |
|  | TC | 74.8% (98) | 80.8% (21) | 1.71 [0.36 – 8.02] |  | 74.8% (83) | 84.6% (22) | 1.85 [0.39 – 8.78] |  |
|  | CC | 13.0% (17) | 11.5% (3) | 1.41 [0.20 – 9.58] |  | 12.6% (14) | 7.7% (2) | 1.00 [0.12 – 8.12] |  |
| rs5945619 |  |  |  |  |  |  |  |  |  |
|  | TT* | 9.1% (12) | 4.0% (1) | 1 | 0.47 | 9.2% (10) | 11.1% (3) | 1 | 0.95 |
|  | TC | 87.1% (115) | 88.0% (22) | 2.29 [0.28 – 18.56] |  | 87.2% (95) | 85.2% (23) | 0.80 [0.06 – 10.59] |  |
|  | CC | 3.8% (5) | 8.0% (2) | 4.80 [0.35 – 65.75] |  | 3.7% (4) | 3.7% (1) | 0.83 [0.20 – 3.17] |  |
| rs620861 |  |  |  |  |  |  |  |  |  |
|  | CC* | 25.7% (36) | 36.0% (9) | 1 | 0.18 | 28.0% (33) | 25.9% (7) | 1 | 0.90 |
|  | CT | 43.6% (61) | 24.9% (6) | 0.39 [0.12 – 1.19] |  | 39.8% (47) | 44.4% (12) | 1.20 [0.42 – 3.38] |  |
|  | TT | 30.7% (43) | 40.0% (10) | 0.93 [0.34 – 2.53] |  | 32.2% (38) | 29.6% (8) | 0.99 [0.32 – 3.03] |  |
| rs6501455 |  |  |  |  |  |  |  |  |  |
|  | GG* | 31.6% (37) | 29.2% (7) | 1 | 0.96 | 30,3% (30) | 26.1% (6) | 1 | 0.17 |
|  | GA | 53.0% (62) | 54.2% (13) | 1.10 [0.40 – 3.02] |  | 51,5% (51) | 69.6% (16) | 1.56 [0.55 – 4.44] |  |
|  | AA | 15.4% (18) | 16.7% (4) | 1.17 [0.30 – 4.53] |  | 18.2% (18) | 4.3% (1) | 0.27 [0.03 – 2.49] |  |
| rs6983267 |  |  |  |  |  |  |  |  |  |
|  | GG* | 8.1% (11) | 0 (0) | 1 | **0.02** | 8.3% (9) | 4.0% (1) | 1 | 0.45 |
|  | GT | 91.5% (118) | 95.8% (23) | 3.14 [ – ] |  | 91.5% (99) | 96.0% (24) | 2.18 [0.26 – 18.06] |  |
|  | TT | 0 (0) | 4.2% (1) | 2.61 [ – ] |  | 0 (0) | 0 (0) | - |  |
| rs6983561 |  |  |  |  |  |  |  |  |  |
|  | AA* | 81.6% (102) | 79.2% (19) | 1 | 0.74 | 80.0% (84) | 91.7% (22) | 1 | 0.38 |
|  | AC | 16.8% (21) | 20.8% (5) | 1.27 [0.42 – 3.80] |  | 18.1% (19) | 8.3% (2) | 0.40 [0.08 – 1.85] |  |
|  | CC | 1.6% (2) | 0 (0) | – |  | 1.9% (2) | 0 (0) | – |  |
| rs7000448 |  |  |  |  |  |  |  |  |  |
|  | GG* | 30.5% (43) | 29.6% (8) | 1 | 0.99 | 32.2% (38) | 29.6% (8) | 1 | 0.42 |
|  | GA | 55.3% (78) | 55.6% (15) | 1.03 [0.40 – 2.63] |  | 51.7% (61) | 63.0% (17) | 1.32 [0.52 – 3.36] |  |
|  | AA | 14.2% (20) | 14.8% (4) | 1.07 [0.28 – 3.99] |  | 16.1% (19) | 7.4% (2) | 0.50 [0.09 – 2.58] |  |
| rs7214479 |  |  |  |  |  |  |  |  |  |
|  | CC* | 0.7% (1) | 0 (0) | 1 | 0.90 | 00.8% (1) | 0 (0) | 1 | 0.43 |
|  | CT | 95.8% (136) | 96.3% (26) | 3.08 [ – ] |  | 94.1% (112) | 100% (27) | 3.89 [ – ] |  |
|  | TT | 3.5% (5) | 3.7% (1) | 3.23 [ – ] |  | 5.0% (6) | 0 (0) | 1.00 [ – ] |  |
| rs7920517 |  |  |  |  |  |  |  |  |  |
|  | AA* | 6.3% (9) | 11.5% (3) | 1 | 0.51 | 9.2% (11) | 3.7% (1) | 1 | 0.58 |
|  | AG | 67.6% (96) | 57.7% (15) | 0.46 [0.11 – 1.93] |  | 66.4% (79) | 66.7% (18) | 2.50 [0.30 – 20.67] |  |
|  | GG | 26.1% (37) | 30.8% (8) | 0.64 [0.14 – 2.97] |  | 24.4% (29) | 29.6% (8) | 3.03 [ 0.33 – 27.15] |  |
| rs7931342 |  |  |  |  |  |  |  |  |  |
|  | GG* | 25.2% (35) | 33.3% (9) | 1 | 0.62 | 28.4% (33) | 14.8% (4) | 1 | 0.21 |
|  | GT | 68.3% (95) | 63.0% (17) | 0.69 [0.28 – 1.70] |  | 63.8% (74) | 81.5% (22) | 2.45 [0.78 – 7.68] |  |
|  | TT | 6.5% (9) | 3.7% (1) | 0.43 [ 0.104 – 3.86] |  | 7.8% (9) | 3.7% (1) | 0.91 [0.09 – 9.25] |  |
| rs983085 |  |  |  |  |  |  |  |  |  |
|  | AA* | 32.9% (46) | 38.5% (10) | 1 | 0.76 | 32.5% (38) | 29.6% (8) | 1 | 0.88 |
|  | AG | 50.0% (70) | 42.3% (11) | 0.72 [0.28 – 1.83] |  | 50.4% (59) | 55.6% (15) | 1.20 [0.46 – 3.12] |  |
|  | GG | 17.1% (24) | 19.2% (5) | 0.95 [0.29 – 3.12] |  | 17.1% (20) | 14.8% (4) | 0.95[0.25 – 3.54] |  |
| rs1859962 |  |  |  |  |  |  |  |  |  |
|  | TT* | 18.7% (26) | 14.8% (4) | 1 | 0.73 | 19.0% (22) | 11.1% (3) | 1 | 0.50 |
|  | TG | 70.5% (98) | 77.8% (21) | 1.39 [0.44 – 4.41] |  | 71.6% (83) | 74.1% (20) | 1.76 [0.48 – 6.49] |  |
|  | GG | 10.8% (15) | 7.4% (2) | 0.86 [0.16 – 3.41] |  | 9.5% (11) | 14.8% (4) | 2.66 [0.50 – 14.06] |  |

*Wild-Type
