# Supplementary material for: Role of Genetic Polymorphisms in the Development and Prognosis of Sporadic and Familial Prostate Cancer
Source: PLoS One. 2016 Dec 1;11(12):e0166380. doi: 10.1371/journal.pone.0166380 (PMC5132395; doi:10.1371/journal.pone.0166380)
Supplement: S2 Table — Genotype frequencies in Gleason <7 and ≥ 7 and Gleason <7, Gleason 7 and Gleason ≥ 8. (DOCX) [file pone.0166380.s002.docx]

**S2 table. Gleason Score**. Genotype frequencies in Gleason <7 and ≥ 7 and Gleason <7, Gleason 7 and Gleason ≥ 8.

| ID SNP | Genotype | Gleason <7 (n) | Gleason ≥7 (n) | Odds Ratio | p | Gleason <7 (n) | Gleason 7 (n) | Gleason ≥8 (n) | p |
| --- | --- | --- | --- | --- | --- | --- | --- | --- | --- |
| rs4242382 |  |  |  |  |  |  |  |  |  |
|  | GG* | 29.7% (11) | 45.0% (45) | 1 | 0.06 | 29.7% (11) | 40.4% (21) | 50.0% (24) | 0.16 |
|  | GA | 8.1% (3) | 15.0% (15) | 1.22 [0.30 – 4.97] |  | 8.1% (3) | 17.3% (9) | 12.5% (6) |  |
|  | AA | 62.2% (23) | 40.0% (40) | 0.42 [0.18 – 0.98] |  | 62.2% (23) | 42.3% (22) | 37.5% (24) |  |
| rs10090154 |  |  |  |  |  |  |  |  |  |
|  | CC* | 87.5% (35) | 80.2 (85) | 1 | 0.48 | 87.5% (35) | 73.1% (38) | 87.0 (47) | 0.15 |
|  | CT | 12.5% (5) | 17.9% (19) | 1.56 [0.54 – 4.52] |  | 12.5% (5) | 23.1% (12) | 0% (0) |  |
|  | TT | 0% (0) | 1.9% (2) | 6.65 [ – ] |  | 0% (0) | 3.8% (2) | 13.0% (7) |  |
| rs1016343 |  |  |  |  |  |  |  |  |  |
|  | CC* | 81.1% (30) | 78.8% (78) | 1 | 0.56 | 81.1% (30) | 74.5% (38) | 83.3% (40) | 0.46 |
|  | CT | 18.9% (7) | 18.2% (18) | 0.98 [0.37 – 2.60] |  | 12,5% (5) | 23.5% (12) | 12.5% (6) |  |
|  | TT | 0% (0) | 3.0% (3) | 6.21 [ – ] |  | 0% (0) | 2.0% (1) | 4.2% (2) |  |
| rs1447295 |  |  |  |  |  |  |  |  |  |
|  | CC* | 36.6% (15) | 52.4% (55) | 1 | 0.13 | 36.6% (15) | 42.6% (23) | 62.7% (32) | 0.07 |
|  | CA | 12.2% (5) | 14.3% (15) | 0.81 [0.25 – 2.61] |  | 12.2% (5) | 18.5% (10) | 9.83% (5) |  |
|  | AA | 51.2% (21) | 33.3% (35) | 0.45 [0.20 – 0.99] |  | 51.2% (21) | 38.9% (21) | 27.5% (14) |  |
| rs16901979 |  |  |  |  |  |  |  |  |  |
|  | CC* | 68.8% (11) | 70.0% (42) | 1 | 0,86 | 68.8% (11) | 61.3% (19) | 79.3% (23) | 0,57 |
|  | CA | 6.3% (1) | 3.3% (2) | 0.52 [0.43 – 6.32] |  | 6.3% (1) | 3.2% (1) | 3.4% (1) |  |
|  | AA | 25.0% (4) | 26.7% (16) | 1.04 [0.29 – 3.77] |  | 25.0% (4) | 35.5% (11) | 17.2% (5) |  |
| rs2660753 |  |  |  |  |  |  |  |  |  |
|  | CC* | 73.2% (30) | 61.3% (68) | 1 | 0.36 | 73.2% (30) | 63.6% (35) | 58.9% (33) | 0.58 |
|  | CT | 24.4% (10) | 33.3% (37) | 1.63 [0.71 – 3.70] |  | 24.4% (10) | 32.7% (18) | 33.9% (19) |  |
|  | TT | 2.4% (1) | 5.4% (6) | 2.64 [0.30 – 22,95] |  | 2.4% (1) | 3.6% (2) | 7.1% (4) |  |
| rs2710646 |  |  |  |  |  |  |  |  |  |
|  | CC* | 24.4% (10) | 46.3% (50) | 1 | 0.50 | 24.4% (10) | 43.4% (23) | 49.1% (27) | 0.14 |
|  | CA | 29.3% (12) | 22.2% (24) | 0.40 [0.15 – 1.05] |  | 29.3% (12) | 29.3% (12) | 23.6% (13) |  |
|  | AA | 46.3% (19) | 31.5% (34) | 0.35 [ 0.14 – 0.86] |  | 46.3% (19) | 35.8% (19) | 27.3% (15) |  |
| rs3760511 |  |  |  |  |  |  |  |  |  |
|  | TT* | 7.5% (3) | 9.1% (10) | 1 | 0.94 | 7.5% (3) | 9.3% (5) | 8.9% (5) | 0.99 |
|  | TG | 85.0% (34) | 82.7% (58) | 0.80 [0.20 – 3.09] |  | 85.0% (34) | 81.5% (44) | 83.9% (47) |  |
|  | GG | 7.5% (3) | 8.2% (9) | 0.90 [0.14 – 5.64] |  | 7.5% (3) | 9.3% (5) | 7.1% (4) |  |
| rs4962416 |  |  |  |  |  |  |  |  |  |
|  | TT* | 10.8% (4) | 12.3% (13) | 1 | 0.92 | 10.8% (4) | 15.4% (8) | 9.3% (5) | 0.77 |
|  | TC | 75.7% (28) | 76.4% (81) | 0.89 [0.26 – 2.95] |  | 75.7% (28) | 71.2% (37) | 81.5% (44) |  |
|  | CC | 13.5% (5) | 11.3% (12) | 0.73 [0.16 – 3.41] |  | 13.5% (5) | 13.5% (7) | 9.3% (5) |  |
| rs5945619 |  |  |  |  |  |  |  |  |  |
|  | TT* | 10.3% (4) | 7.7% (4) | 1 | 0.75 | 10.3% (4) | 6.0% (3) | 9.3% (5) | 0.51 |
|  | TC | 87.2% (34) | 4.8% (5) | 1.33 [0.37 – 4.73] |  | 87.2% (34) | 86.0% (43) | 88.9% (48) |  |
|  | CC | 2.6% (1) | 87.5% (91) | 2.50 [0.21 – 29.25] |  | 2.6% (1) | 8.0% (4) | 1.9% (1) |  |
| rs620861 |  |  |  |  |  |  |  |  |  |
|  | CC* | 33.3% (14) | 27.5% (30) | 1 | 0.78 | 33.3% (14) | 18.5% (10) | 36.4% (20) | 0.29 |
|  | CT | 38.1% (16) | 41.3% (45) | 1.31 [0.55 – 3.08] |  | 38.1% (16) | 44.4% (24) | 38.2% (21) |  |
|  | TT | 28.6% (12) | 31.2% (34) | 1.32 [ 0.53 – 3.29] |  | 28.6% (12) | 37.0% (20) | 25.5% (14) |  |
| rs6501455 |  |  |  |  |  |  |  |  |  |
|  | GG* | 27.8% (10) | 31.5% (29) | 1 | 0.61 | 27.8% (10) | 25.0% (11) | 37.5% (18) | 0.52 |
|  | GA | 61.1% (22) | 52.2% (48) | 0.75 [0.31 – 1.81] |  | 61.1% (22) | 54.5% (24) | 50.0% (24) |  |
|  | AA | 11.1% (4) | 16.3% (15) | 1.29 [0.34 – 4.82] |  | 11.1% (4) | 20.5% (9) | 12.5% (6) |  |
| rs6983267 |  |  |  |  |  |  |  |  |  |
|  | GG* | 14.3% (5) | 4.8% (5) | 1 | 0.14 | 14.3% (5) | 5.6% (3) | 3.9% (2) | 0.25 |
|  | GT | 85.7% (30) | 94.3% (99) | 3.30 [0.89 – 12.17] |  | 85.7% (30) | 94.4% (51) | 94.1% (48) |  |
|  | TT | 0% (0) | 1.0% (1) | 1.61 [ – ] |  | 0% (0) | 0% (0) | 2.0% (1) |  |
| rs6983561 |  |  |  |  |  |  |  |  |  |
|  | AA* | 79.4% (27) | 81.2% (82) | 1 | 0.22 | 79.4% (27) | 83.3% (40) | 79.2% (42) | 0.51 |
|  | AC | 17.6% (6) | 18.8% (19) | 1.04 [0.37 – 2.87] |  | 17.6% (6) | 16.7% (8) | 20.8% (11) |  |
|  | CC | 2.9% (1) | 1.% (0) | – |  | 2.9% (1) | 0% (0) | 0% (0) |  |
| rs7000448 |  |  |  |  |  |  |  |  |  |
|  | GG* | 23.8% (10) | 35.1% (39) | 1 | 0.28 | 23.8% (10) | 40.0% (22) | 30.4% (17) | 0.30 |
|  | GA | 57.1% (24) | 53.2% (59) | 0.63 [0.27 – 1.46] |  | 57.1% (24) | 52.7% (29) | 53.6% (30) |  |
|  | AA | 19.0% (8) | 11.7% (13) | 0.41 [0.13 – 1.27] |  | 19.0% (8) | 7.3% (4) | 16.1% (9) |  |
| rs7214479 |  |  |  |  |  |  |  |  |  |
|  | CC* | 0% (0) | 0% (0) | 1 | 0.20 | 0% (0) | 0% (0) | 0% (0) | 0.39 |
|  | CT | 92.9% (39) | 97.3% (109) | – |  | 92.9% (39) | 92.9% () | 96.4% (54) |  |
|  | TT | 7.1% (3) | 2.7% (3) | 2.79 [0.54 – 14.43] |  | 7.1% (3) | 1.8% (1) | 3.6% (2) |  |
| rs7920517 |  |  |  |  |  |  |  |  |  |
|  | AA* | 2.4% (1) | 9.9% (11) | 1 | 0.30 | 2.4% (1) | 9.1% (5) | 10.7% (6) | 0.63 |
|  | AG | 69.0% (29) | 64.0% (71) | 0.22 [0.02 – 1.80] |  | 69.0% (29) | 65.5% (36) | 26.8% (15) |  |
|  | GG | 28.6% (12) | 26.1% (29) | 0.22 [0.25 – 1.89] |  | 28.6% (12) | 25.5% (14) | 62.5% (35) |  |
| rs7931342 |  |  |  |  |  |  |  |  |  |
|  | GG* | 29.3% (12) | 25.5% (28) | 1 | 0.13 | 29.3% (12) | 33.3% (18) | 17.9% (10) | **0.02** |
|  | GT | 70.7% (29) | 65.5% (72) | 1.06 [0.47 – 2.37] |  | 70.7% (29) | 63.0% (34) | 67.9% (38) |  |
|  | TT | 0 (0) | 9.1% (10) | - |  | 0 (0) | 3.7% (2) | 14.3% (8) |  |
| rs983085 |  |  |  |  |  |  |  |  |  |
|  | AA* | 36.6% (15) | 31.8% (35) | 1 | 0.37 | 36.6% (15) | 37.0% (20) | 26.8% (15) | 0.07 |
|  | AG | 58.5% (24) | 45.5% (50) | 0.89 [0.41 – 1.94] |  | 58.5% (24) | 44.4% (24) | 46.4% (26) |  |
|  | GG | 4.9% (2) | 22.7% (19) | 5.35 [1.12 – 25.54] |  | 4.9% (2) | 18.5% (10) | 26.8% (15) |  |
| rs1859962 |  |  |  |  |  |  |  |  |  |
|  | TT* | 14.6% (6) | 15.5% (17) | 1 | 0.96 | 14.6% (6) | 16.7% (9) | 14.3% (8) | 0.97 |
|  | TG | 75.6% (31) | 73.6% (81) | 0.92 [0.33 – 2.55] |  | 75.6% (31) | 74.1% (40) | 73.2% (41) |  |
|  | GG | 9.8% (4) | 10.9% (12) | 1.05 [0.24 – 4.58] |  | 9.8% (4) | 9.3% (5) | 12.5% (7) |  |

*Wild-Type
