# Supplementary material for: Role of Genetic Polymorphisms in the Development and Prognosis of Sporadic and Familial Prostate Cancer
Source: PLoS One. 2016 Dec 1;11(12):e0166380. doi: 10.1371/journal.pone.0166380 (PMC5132395; doi:10.1371/journal.pone.0166380)
Supplement: S3 Table — Allele and genotype frequencies according to biochemical Recurrence (DOCX) [file pone.0166380.s003.docx]

**S3 table. Recurrence.** Allele and genotype frequencies according to biochemical recurrence

| ID SNP | Genotype | Without recidive (n) | Recidive (n) | Odds Ratio | p | allele | Without recidive (n) | Recidive (n) | Odds Ratio | p |
| --- | --- | --- | --- | --- | --- | --- | --- | --- | --- | --- |
| rs4242382 |  |  |  |  |  |  |  |  |  |  |
|  | GG* | 36.1% (39) | 54.8% (17) | 1 | 0.15 | G* | 70.2% (40) | 82.9% (68) | 1.00 | 0.07 |
|  | GA | 13.9% (15) | 12.9% (4) | 0.61 [0.17 – 2.11] |  | A | 29.8% (17) | 17.1% (14) | 0.48 [0.21 – 1.08] |  |
|  | AA | 50.0% (54) | 32.3% (10) | 0.42 [0.17 – 1.02] |  |  |  |  |  |  |
| rs10090154 |  |  |  |  |  |  |  |  |  |  |
|  | CC* | 78.9% (90) | 90.9% (30) | 1 | 0.27 | C* | 75.2% (91) | 88.5% (23) | 1.00 | 0.14 |
|  | CT | 19.3% (22) | 9.1% (3) | 0.40 [0.11 – 1.46] |  | T | 24.8% (30) | 11.5% (3) | 0.39 [0.11 – 1.41] |  |
|  | TT | 1.8% (2) | 0 (0) | – |  |  |  |  |  |  |
| rs1016343 |  |  |  |  |  |  |  |  |  |  |
|  | CC* | 79.1% (87) | 80.8% (21) | 1 | 0.52 | C* | 81.3% (87) | 79.3% (23) | 1.00 | 0.80 |
|  | CT | 16.4% (18) | 20.8% (5) | 1.15 [0.38 – 3.45] |  | T | 18.7% (22) | 20.7% (6) | 1.13 [0.40 – 3.15] |  |
|  | TT | 4.5% (5) | 12.5% (3) | – |  |  |  |  |  |  |
| rs1447295 |  |  |  |  |  |  |  |  |  |  |
|  | CC* | 42.7% (50) | 71.0% (22) | 1 | **0.01** | C* | 69.4% (50) | 88.2% (67) | 1.00 | **0,00** |
|  | CA | 17.9% (21) | 6.5% (2) | 0.21 [0.04 – 1.00] |  | A | 30.6% (22) | 11.8% (9) | 0.30 [0.12 – 0.72] |  |
|  | AA | 39.3% (46) | 22.6% (7) | 0.34 [0.13 – 0.88] |  |  |  |  |  |  |
| rs16901979 |  |  |  |  |  |  |  |  |  |  |
|  | CC* | 66.2% (47) | 77.8% (17) | 1 | 0,06 | C* | 74.1% (40) | 83.3% (20) | 1.00 | 0,37 |
|  | CA | 1.7% (1) | 11.1% (2) | 5.71 [0.48 – 67.99] |  | A | 25.9% (14) | 16.7% (4) | 0.57 [0.16 – 1.96] |  |
|  | AA | 31.7% (19) | 11.1% (2) | 0.30 [0.06 – 1.45] |  |  |  |  |  |  |
| rs2660753 |  |  |  |  |  |  |  |  |  |  |
|  | CC* | 60.8% (73) | 66.7% (22) | 1 | 0.74 | C* | 76.8% (73) | 81.0% (47) | 1.00 | 0.54 |
|  | CT | 33.3% (40) | 30.3% (10) | 0.83 [0.35 – 1.92] |  | T | 23.2% (22) | 19.0% (11) | 0.77 [0.34 – 1.74] |  |
|  | TT | 5.8% (7) | 3.0% (1) | 0.47 [0.05 – 4.06] |  |  |  |  |  |  |
| rs2710646 |  |  |  |  |  |  |  |  |  |  |
|  | CC* | 36.4% (43) | 48.5% (16) | 1 | 0.45 | C* | 72.9% (43) | 81.5% (75) | 1.00 | 0.21 |
|  | CA | 27.1% (32) | 21.2% (7) | 0.58 [0.21 – 1.59] |  | A | 27.1% (16) | 18.5% (17) | 0.60 [0.28 – 1.32] |  |
|  | AA | 36.4% (43) | 30.3% (10) | 0.62 [ 0.25 –1.53] |  |  |  |  |  |  |
| rs3760511 |  |  |  |  |  |  |  |  |  |  |
|  | TT* | 8.3% (10) | 3.1% (1) | 1 | 0.53 | T* | 90.0% (9) | 78.2% (111) | 1.00 | 0.37 |
|  | TG | 83.3% (100) | 90.6% (29) | 2.90 [0.35 – 23.60] |  | C | 10.0% (1) | 21.8% (31) | 2.51 [0.30 – 20,60] |  |
|  | GG | 8.3% (10) | 6.3% (2) | 2.00 [0.15 – 25.75] |  |  |  |  |  |  |
| rs4962416 |  |  |  |  |  |  |  |  |  |  |
|  | TT* | 11.6% (13) | 15.2% (5) | 1 | 0.53 | T* | 72,2% (13) | 78.0% (99) | 1.00 | 0.58 |
|  | TC | 75.9% (85) | 78.8% (26) | 0.79 [0.25 – 2.44] |  | C | 27,8% (5) | 22.0% (28) | 0.73 [0.24 – 2.23] |  |
|  | CC | 12.5% (14) | 6.1% (2) | 0.37 [0.06 – 2.25] |  |  |  |  |  |  |
| rs5945619 |  |  |  |  |  |  |  |  |  |  |
|  | TT* | 10.6% (12) | 3.0% (1) | 1 | 0.12 | T* | 92.3% (12) | 75.9% (101) | 1.00 | 0.17 |
|  | TC | 86.7% (98) | 87.9% (29) | 3.55 [0.447 – 28.47] |  | C | 7.7% (1) | 24.1% (32) | 3.80 [0.47 – 30.38] |  |
|  | CC | 2.7% (3) | 9.1% (3) | 12.00 [0.89 – 160.40] |  |  |  |  |  |  |
| rs620861 |  |  |  |  |  |  |  |  |  |  |
|  | CC* | 27.5% (33) | 30.3% (10) | 1 | 0.56 | C* | 76.7% (33) | 79.1% (87) | 1.00 | 0.75 |
|  | CT | 43.3% (52) | 33.3% (11) | 0.69 [0.26 – 1.82] |  | T | 23.3% (10) | 20.9% (23) | 0.87 [0.37 – 2.02] |  |
|  | TT | 29.2% (35) | 36.4% (12) | 1.13 [0.43 – 2.96] |  |  |  |  |  |  |
| rs6501455 |  |  |  |  |  |  |  |  |  |  |
|  | GG* | 33.7% (33) | 25.8% (8) | 1 | 0.68 | G* | 80.5% (33) | 73.9% (65) | 1.00 | 0.41 |
|  | GA | 51.0% (50) | 54.8% (17) | 1.40 [0.54 – 3.62] |  | A | 19.5% (8) | 26.1% (23) | 1.46 [0.58 – 3.61] |  |
|  | AA | 15.3% (15) | 19.4% (6) | 1.65 [0.48 – 5.59] |  |  |  |  |  |  |
| rs6983267 |  |  |  |  |  |  |  |  |  |  |
|  | GG* | 6.4% (7) | 12.1% (4) | 1 | 0,10 | G* | 50.0% (3) | 77.9% (106) | 1.00 | 0.11 |
|  | GT | 93.6% (102) | 84.8% (28) | 0.48 [0.13 – 1,75] |  | T | 50.0% (3) | 22.1% (30) | 0.28 [0.05 – 1.47] |  |
|  | TT | 0 (0) | 3.0% (1) | 2.82 [ – ] |  |  |  |  |  |  |
| rs6983561 |  |  |  |  |  |  |  |  |  |  |
|  | AA* | 83.0% (882) | 75.0% (24) | 1 | 0.47 | A* | 79.3% (88) | 66.7% (18) | 1.00 | 0.16 |
|  | AC | 16.0% (17) | 21.9% (7) | 1.51 [0.56 – 4.06] |  | C | 20.7% (23) | 33.3% (9) | 1.91 [0.76 – 4.81] |  |
|  | CC | 0.9% (1) | 3.1% (1) | 3.66 [ 0.22 – 60.80] |  |  |  |  |  |  |
| rs7000448 |  |  |  |  |  |  |  |  |  |  |
|  | GG* | 31.7% (38) | 26.5% (9) | 1 | 0.56 | G* | 80.9% (38) | 76.6% (82) | 1.00 | 0.56 |
|  | GA | 51.7% (62) | 61.8% (21) | 1.43 [0.59 – 3.44] |  | A | 19.1% (9) | 23.4% (25) | 1.28 [0.54 – 3.02] |  |
|  | AA | 16.7% (20) | 11.8% (4) | 0.84 [0.23 – 3.08] |  |  |  |  |  |  |
| rs7214479 |  |  |  |  |  |  |  |  |  |  |
|  | CC* | 0 (0) | 2.9% (1) | 1 | 0.16 | C* | 0 (0) | 78.6% (121) | 1.00 | **0.05** |
|  | CT | 95.9% (116) | 94.1% (32) | - |  | T | 100% (1) | 21.4% (33) | * |  |
|  | TT | 4.1% (5) | 2.9% (1) | - |  |  |  |  |  |  |
| rs7920517 |  |  |  |  |  |  |  |  |  |  |
|  | AA* | 7.4% (9) | 8.8% (3) | 1 | 0.93 | A* | 75.0% (9) | 78.3% (112) | 1.00 | 0.78 |
|  | AG | 63.6% (77) | 64.7% (22) | 0.85 [0.21 – 3.44] |  | G | 25.0% (3) | 21.7% (31) | 0.83 [0.21 – 3.25] |  |
|  | GG | 28.9% (35) | 26.5% (9) | 0.77 [0.17 – 3.45] |  |  |  |  |  |  |
| rs7931342 |  |  |  |  |  |  |  |  |  |  |
|  | GG* | 28.0% (33) | 20.6% (7) | 1 | 0.42 | G* | 82.5% (33) | 75.9% (85) | 1.00 | 0.38 |
|  | GT | 65.3% (77) | 76.5% (26) | 1.59 [0.62 – 4.03] |  | T | 17.5% (7) | 24.1% (27) | 1.49 [0.59 – 3.77] |  |
|  | TT | 6.8% (8) | 2.9% (1) | 0.58 [0.06 – 5.49] |  |  |  |  |  |  |
| rs983085 |  |  |  |  |  |  |  |  |  |  |
|  | AA* | 31.9% (38) | 33.3% (10) | 1 | 0.98 | A* | 77.6% (38) | 78.6% (81) | 1.00 | 0.87 |
|  | AG | 50.4% (60) | 48.5% (16) | 0.92 [0.38 – 2.19] |  | G | 22.4% (11) | 21.4% (22) | 0.93 [0.41 – 2.13] |  |
|  | GG | 17.6% (21) | 18.2% (5) | 0.98 [ 0.31 – 3.05] |  |  |  |  |  |  |
| rs1859962 |  |  |  |  |  |  |  |  |  |  |
|  | TT* | 19.3% (23) | 11.8% (4) | 1 | 0.14 | T* | 85.2% (23) | 76.2% (96) | 1.00 | 0.30 |
|  | TG | 71.4% (85) | 67.6% (23) | 1.55 [0.48 – 4.95] |  | G | 14.8% (4) | 23.8% (30) | 1.79 [0.57 – 5.60] |  |
|  | GG | 9.2% (11) | 20.6% (7) | 3.65 [0.88 – 15.18] |  |  |  |  |  |  |

*Wild-Type
